# Using directional OCT to analyze photoreceptor visibility over AMD-related drusen

**DOI:** 10.1038/s41598-022-13106-3

**Published:** 2022-06-13

**Authors:** Brennan Marsh-Armstrong, Kelly S. Murrell, Denise Valente, Ravi S. Jonnal

**Affiliations:** University of California, Davis Eye Center, Sacramento, USA

**Keywords:** Biomedical engineering, Diagnostic markers, Retinal diseases, Optical imaging

## Abstract

Investigators have reported reduced visibility of the cone photoreceptors overlying drusen using adaptive optics (AO) imaging techniques. Two hypotheses have been proposed to explain this phenomenon. First, the disease-related deformation of the photoreceptor outer segment (OS) may reduce its ability to act as a wave guide, thus decreasing the cell’s familiar reflectance pattern. Second, drusen could disorient the photoreceptors away from the eye’s pupil, reducing the amount of light reflected back out the pupil. In this work, we use directional OCT (dOCT) images of drusen in AMD patients to measure the respective contributions of these deforming and disorienting factors.

## Introduction

Age-related macular degeneration (AMD) affects 6.5% of Americans over the age of 40^1^, over 10 million people in the United States alone. The annual global cost of treating the disease likely exceeds $300 billion, a sum that could double by 2050^2^. While anti-VEGF medications are a standard treatment for wet AMD^3^, recent evidence suggests that they may not delay the underlying atrophy^4^. Moreover, the more common dry form of the disease has no accepted treatment. Fortunately, there is promising research on the development of pharmaceuticals, stem cell technologies^5,6^, and gene therapies^7^ to treat the disease. This is especially exciting given evidence of intact inner retinal neurons in AMD^8^. The developments of these novel therapeutics depend on the existence of reliable biomarkers of disease progression.

One of the hallmarks of AMD is the presence of large ($>125\;\upmu {\mathrm{m}}$) accumulations of lipids and bisretinoids between the retinal pigmented epithelium (RPE) and Bruch’s membrane (BM), which are known as drusen. Drusen result in local topographical disruptions of the outer retina, leading to ostensible disorientation of the photoreceptor layer. In healthy retinae, the optical axis of the photoreceptor is approximately normal to the underlying RPE and BM layers. Above drusen, by contrast, photoreceptors appear to be splayed outward, oriented normal to the curved surface of the druse.

Over the past two decades, the development of adaptive optics (AO) retinal imaging modalities has led to visualization of retinal structure and function with unprecedented resolution^9–11^. The structure and function of cones in particular have been studied in great detail. Since vision loss in AMD is mediated by the loss of photoreceptors, investigators have employed AO to study the impacts of the disease at the cellular level. While improved tools for quantification and identification of lesions has has been gained from these efforts^12^, quantitative analysis of the cones at the cellular level—e.g., their spatial distribution or cellular morphometry—has been limited^13–17^.

Cones overlying drusen, in particular, have been difficult to visualize. Those cells are well-known for having strong directionality, where light entering the eye at the center of the pupil, hitting the retina at small angles, produces a stronger photoreceptor response compared to light of equal intensity entering near the edge of the pupil. This angular selectivity, referred to as the psychophysical Stiles–Crawford effect (SCE)^18^, is also observed in light backscattered by the retina, the optical Stiles–Crawford effect (OSCE)^19^. This leads to speculation whether the photoreceptors overlying the drusen have suffered structural damage leading to less reflectivity or whether their reduced visibility could be due to their pointing away from the eye’s optical axis and the pupil. Both of these hypotheses are consistent with observations that retinal sensitivity is reduced in parts of the macula where drusen are present^20^.

Directional optical coherence tomography (dOCT) is a method by which the angle between the imaging beam (and light detection path) and the retina is varied by translating the subject’s eye laterally in directions perpendicular to the beam. While the directional images are typically acquired in series, parallel detection has been demonstrated using multiple beams in scanning spectral-domain OCT^21^ and carrier multiplexing in full-field swept-source OCT^22^. Directional OCT has been used to measure the OSCE and characterize the directionality of neural layers in the outer retina^23^. It has also been used to visualize the Henle fiber layer (HFL), which scatters light preferentially toward the opposite side of the eye’s pupil^24,25^. The improved contrast of HFL permits its disambiguation from the apposed outer nuclear layer (ONL)^26^. Reliable measurement of ONL thickness is important because thinning of ONL is a sign of photoreceptor loss^27,28^. Disease-related changes in directional scattering have been reported in several layers of the neural retina, and a detailed approach for measuring these changes using clinical OCT systems has been demonstrated^29^. A similar approach has also been used in mouse models to distinguish the scattering profiles of RPE melanin^30^ and to measure pigmentary changes in a mouse model of Stargardt’s disease^31^.

More recently, Griffin et al. used dOCT to study changes in the reflectivity of the inner–outer segment junction (IS/OS), also called the ellipsoid zone (EZ), caused by dry AMD^32^. It was determined that IS/OS amplitude was highest in normal subjects, reduced in the apparently unaffected portions of AMD retinae, and further reduced above drusen. The investigators showed that by offsetting the beam in the pupil, IS/OS amplitude was reduced in all three cases.

Clinical images of AMD drusen show reflectivity changes in the outer retinal bands, hypothetically attributed either to cone loss or changes in orientation, respectively resulting in angle-independent and angle-dependent changes in reflectivity. The purpose of this study is to quantify the effect of drusen on directionality and other optical properties of cones. We will test the hypothesis that reduced visibility of cones is mutlifactorial by using directional OCT to determine the relative contributions of directional and nondirectional factors. Unlike other studies of photoreceptor directionality and the SCE, we did not monitor the position of the beam in the pupil but rather inferred the effective pupil position from the angle of illumination. A consequence of this approach is that when the imaging beam is centered in the pupil, the IS/OS band on the slopes of the druse have decentered effective pupil positions. Those portions of the IS/OS have effectively centered imaging when the beam is near the edges of the pupil. Thus, if disorientation of the cones overlying drusen were the primary cause for their reduced visibility, the directionality of those cones would be very similar to their non-drusen counterparts.

## Methods

### Imaging apparatus

Imaging was performed with a custom, spectrometer-based scanning SD-OCT system constructed in our lab. A superluminescent diode (SLD) (Superlum Ltd., Cork, Ireland; $\lambda =855\;{\mathrm{nm}}$; $\Delta \lambda =75\; {\mathrm{nm}}$) was scanned over the retina using a galvonometer scanner. Although the system has two scanners for volumetric imaging, only the horizontal scanner was used in this study. Its axial and lateral resolutions were approximately 4.5 $\upmu {\mathrm{m}}$ and 12 $\upmu {\mathrm{m}}$ in the eye ($n=1.38$). The fiber-based Michelson interferometer incorporated an 80/20 beam splitter. The power of the imaging beam at the eye was measured to be between 625 and 650 $\upmu {\mathrm{W}}$ for all imaging sessions, below the maximum permissible exposure determined by ANSI^33^. The OCT signal was detected by a custom spectrometer consisting of reflective diffraction grating (1200 ${\mathrm{mm}}^{-1}$) and CMOS line-scan camera (spL4096-140 km; Basler, Ahrensburg, Germany). The camera was operated at a rate of 125 kHz, which determined the system’s A-scan acquisition rate. The horizontal galvo scanned over a 2 mm field of view in a sawtooth pattern, acquiring 400 A-scans in each B-scan, at a rate of 313 B-scans/s.

### Patient recruitment and imaging

Four subjects with intermediate AMD and isolated drusen, graded by clinicians in the UC Davis Eye Center using the Age-related Eye Disease Study (AREDS) criteria, were recruited for participation in the study. To avoid intersubject variability due to eccentricity, which is known to affect cone directionality^23^, candidate drusen were required to lie in an annular band between $6^\circ$ and $8^\circ$ of the foveal center. Drusen were identified in clinical OCT images and/or fundus photos. Written informed consent was acquired from each subject, as specified in the University of California, Davis Institutional Review Board approval (UC Davis IRB #1298234-10). The study adhered to the tenets of the Declaration of Helsinki. Prior to imaging, subjects were cyclopleged and dilated using one drop of tropicamide 1% and one drop of phenylephrine 2.5%. A bite bar and a forehead rest were employed to position and stabilize the subject’s pupil during imaging. Subject fixation was guided with a calibrated target. During dilation, the druse (n) of interest were located in the OCT image through fine adjustment of the fixation target. Once the druse (n) were located, further fine adjustment of the fixation target was used to position the beam on the tallest part of the druse. In the plane of the B-scan, the druse was positioned in such a away that apparently healthy retina occupied most of the B-scan.

Once the imaging beam was positioned on the subject’s retina, imaging proceeded as follows. The subject was aligned to the system such that the imaging beam was vertically centered in the pupil. The subject was then translated horizontally such that the beam passed through one edge of their pupil. Series of between 1600 and 2400 B-scans were acquired at each of 8–12 locations in the pupil, starting at one edge of the pupil and spaced 0.64 mm apart. The number of locations was dictated by the dilated pupil diameter. Imaging was concluded when the beam was clipped by the opposite edge of the pupil.

### Image processing and analysis

Spectral data from the OCT system were post-processed as follows. First, acquired spectral fringes were mapped into wave number (*k*) space using linear interpolation. Next, dispersion mismatch between the sample and reference arms of the interferometer was corrected using a numerical optimization approach. Then fringes were converted into A-scans using discrete Fourier transformation (DFT) with zero-padding to oversample by a factor of two in the axial dimension. The resulting B-scans were also oversampled in the lateral dimension by a factor of two, using nearest-neighbor interpolation.

B-scans were aligned and averaged using a custom semi-rigid body algorithm as follows. A reference frame was selected by cross-correlating a random subset of 100 B-scans with one another and selecting one that bore maximum correlation with the rest of the subset. The full series of 1600–2400 target B-scans was then registered to this reference image. Whole target B-scans were aligned to the reference, and then subdivided into eight strips, each of which was realigned separately. This step was done to correct axial movement artifacts ocurring within a single scan. All image registration was done using DFT-based cross-correlation after normalization^34^. Correlation of the aligned images (or, equivalently, cross-correlation maxima) were recorded during this process, and only B-scans exceeding a correlation threshold were averaged. For each subject, a threshold was selected which would permit registration of approximately 50 B-scans. Correlation thresholds varied between 0.6 and 0.7, presumably due to variations in image signal-to-noise ratio (SNR) and/or fixation stability. In the resulting averaged images, a semi-automated method was used to segment the IS/OS band. The cone outer segment (COST) band was not studied because a clear boundary between COST and retinal pigmented epithelium (RPE) was rarely observed above drusen, and contamination by light scattered from RPE was unavoidable.

**Figure 1:**
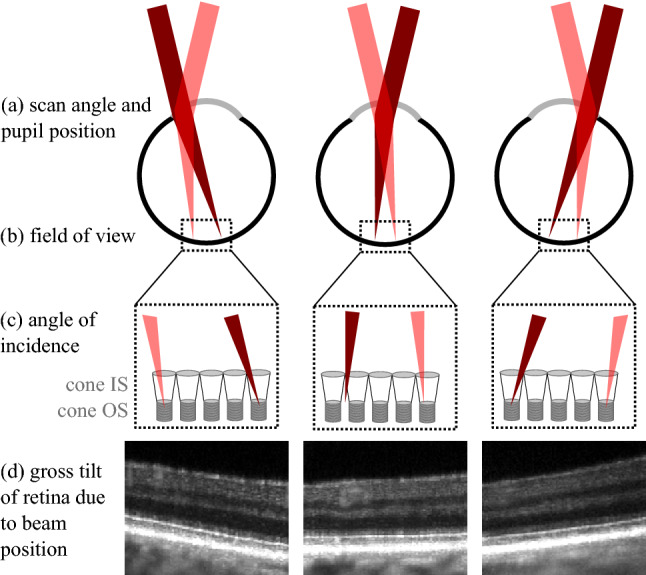
Concept of directional OCT. (**a**) To measure direction-dependent changes in the OCT image, the scanning beam is moved across the pupil. The light red and dark red beams represent the extremes of the scan angle. The pupil of the eye is optically conjugated with the galvo scanners, and, thus, the beam does not move in the pupil while scanning. (**b**) Notwithstanding eye movements, the portion of retina scanned is constant with respect to the beam’s position in the pupil. (**c**) The angle with which the beam illuminates the retina, however, changes as the beam is moved in the pupil. Additional angular differences may arise over the course of the scan if the scanner/pupil plane is displaced from the eye’s optical center. (**d**) When the beam is moved in the pupil, the OCT B-scan appears to tilt due to scan-angle-dependent variation in the beam’s path length.

When the OCT scanners are conjugated with the optical center of the eye, both the optical path length (OPL) between the OCT reference mirror and the retina and the angle of illumination (AOI) are constant with respect to scan angle. When the eye is displaced from this location in any direction, the system creates scan-angle-dependent variations in OPL and AOI, as shown in Fig. 1. In particular, when the beam is moved to one side of the pupil, the ipsilateral side of the retina is moved closer to the scanners, while the contralateral side of the retina is moved further, as shown in Fig. 1 (top). The angle-dependent variation in OPL explains the apparent tilt of the retina as the subject’s pupil is moved with respect to the beam. In addition to the gross change in AOI, displacement of the scanner conjugate from the eye’s center of curvature generates scan-angle-dependent variations in AOI. Both of these sources of AOI diversity are depicted in Fig. 1 (center). If the position of the beam in the pupil and eye length are known, the AOI can be approximated, but variations in retinal thickness and/or curvature cause some error. Instead of calculating the AOI based on the beam position and eye length, we chose to measure the AOI directly from the OCT image. In a B-scan, the direction of light propagation is identical to the A-scan orientation (i.e., vertical). Thus, the AOI was extracted directly from the OCT B-scans by calculating the local tangent of a given surface or feature in the B-scan.

For each A-scan in each B-scan, the AOI and amplitude of the IS/OS band (integrated over a $3 \times 3$ pixel window) were measured. To facilitate comparison between results of our Gaussian modeling and prior studies of photoreceptor directionality, we then calculated an effective pupil position, using a standard eye focal length of 16.7 mm. The Gaussian modeling of IS/OS amplitude could be done directly on the AOI, but instead we used effective pupil position in order to make the resulting estimates of directionality commensurate with earlier work.

To control for angle-dependent variation in overall retinal reflectance (due to scan-angle-dependent factors including variations in media opacity or optical transmission in the eye or imaging system), we normalized the IS/OS amplitude by the overlying outer nuclear layer (ONL), a part of the B-scan thought to be isotropically scattering. Hereafter, only the normalized amplitude of the IS/OS is considered.

### Determining the effect of drusen on IS/OS amplitude and directionality

First, we sought to determine whether the IS/OS amplitude differed between the non-drusen and drusen zones. Using data from individual subjects and pooled among subjects, we performed unpaired t-tests comparing the amplitude of non-drusen IS/OS and drusen IS/OS. To improve visualization and fitting of the data, we next employed a rolling average to smooth the data. A window with an effective width of 2 mm was shifted in 1 mm increments, while binning the normalized IS/OS amplitude accordingly. The mean and median of the resulting bins were computed. Next, for each subject, the effective pupil position *x* and smoothed IS/OS amplitude $a_I(x)$ were fit with a four-parameter Gaussian model, separately for non-drusen and drusen zones:

1 $$\begin{aligned} a_I(x) = B + A \times 10^{-\rho [x-x_0]}, \end{aligned}$$

Fitting was performed using a Levenberg–Marquardt least-squares minimization, and resulting values of *B*, *A*, and $\rho$ were recorded for each subject. $x_0$ was a free parameter in the fitting procedure but not considered in further analysis. The resulting values were used to visualize the directionality profiles of the non-drusen and drusen zones in all four subjects and tested for statistically significant differences between zones by pooling the values among subjects.

Early efforts to model the SCE with a Gaussian function used only three parameters, corresponding to our *A*, $\rho$, and $x_0$^35^. The good fit achieved by that approach suggests that sensitivity, measured psychophysically, goes to zero at large angles of incidence. However, investigators studying the OSCE recognized that an additional term was useful for capturing an isotropic scattering component^36^. In our own measurements, fitting error was greatly improved by including this parameter, suggesting that OCT is sensitive to this component as well and echoing earlier measurements of the OSCE using OCT^23^.

## Results

### B-scan images and segmentation

**Figure 2:**
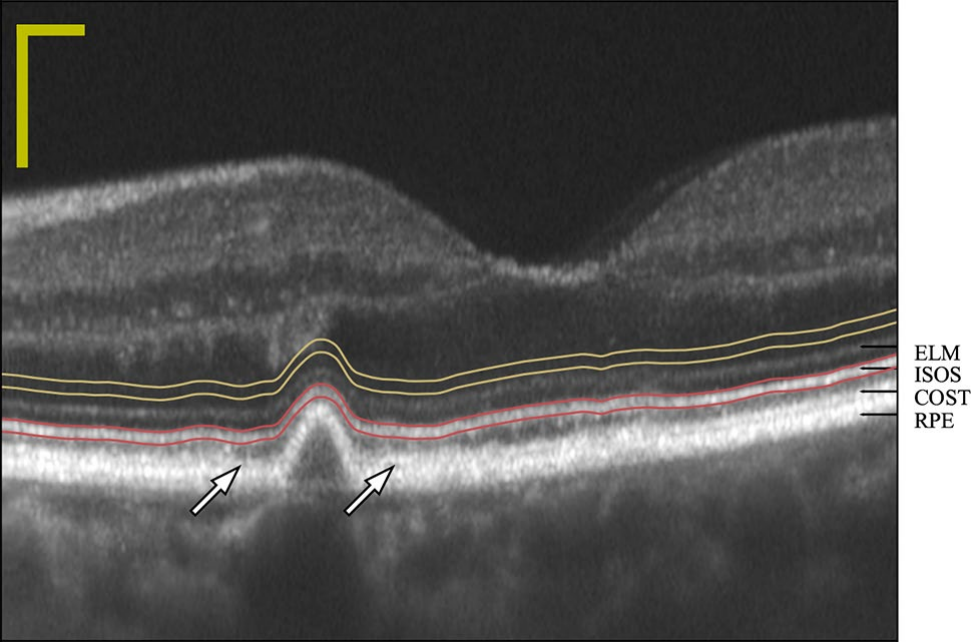
Example B-scan with segmented regions. An example of a B-scan from subject 2, shown in decibel (dB) scale. Dynamic ranges of images varied with pupil position and subject, with most B-scans falling between 30 and 45 dB. An isolated intermediate druse ($100\, \upmu {\mathrm{m}}$) is visible, approximately $1.5^\circ$ nasal to the fovea. The IS/OS band was identified using a semi-automated procedure. The resulting trace through the band is indicated with two red lines, and the nominal depth of the inner-outer segment junction (IS/OS) is the point between those lines. The amount of light (amplitude) scattered by the IS/OS band is the vertical sum of the pixels lying between the red lines. IS/OS amplitude was normalized by dividing its value by that of a presumably isotropic scattering region in the outer nuclear layer (ONL), indicated here with two yellow lines. In healthy parts of most retinae, at least four outer retinal bands are visible, likely originating from the external limiting membrane (ELM), IS/OS, cone outer segment tips (COST), and retinal pigmented epithelium (RPE), labeled at the right edge of the image. Above the druse, only three bands are visible—ELM, IS/OS, and a third, broader band, which separates into COST and RPE (white arrows) at the interface with the non-drusen zone. Scale bars indicate $100\, \upmu {\mathrm{m}}$ in each dimension.

In all four subjects, averaged B-scans included drusen and adjacent, apparently unaffected retina. The overall tilt of the B-scan appeared to correspond to pupil entry position, as expected. In the non-drusen zone of each image, four outer retinal bands were evident: the external limiting membrane (ELM), IS/OS, COST, and retinal pigmented epithelium (RPE), as evident in Fig. 2. In the druse-affected portions of the retina, only ELM and IS/OS were clearly distinguishable. Distal to those, a broader, more diffuse band was visible, which divided into two bands (COST and RPE) at the edges of the druse, as indicated by the white arrows in Fig. 2. The segmented IS/OS band is indicated by red lines in Fig. 2, while the portion of the ONL selected for normalization is indicated by yellow lines. The normalization region appeared to have uniform amplitude and relatively low angle-dependent variation in amplitude.

### Gross differences were observed between non-drusen and drusen IS/OS amplitude

**Figure 3:**
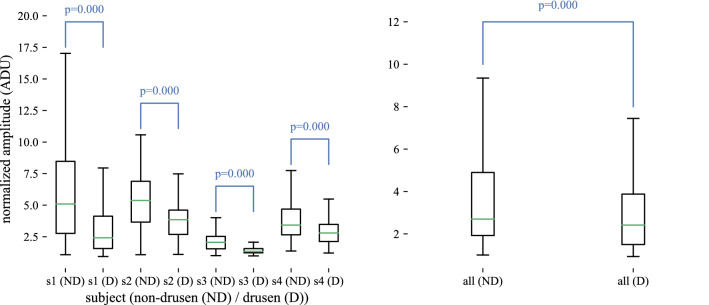
Gross differences between IS/OS amplitude of the two zones. (Left) IS/OS amplitude $a_I$ as a function of subject and zone. Within each subject, significant differences were found in $a_I$ between drusen and non-drusen zones ($p<10^{-3}$), with the apparently healthy IS/OS having higher amplitude in all subjects than IS/OS overlying drusen. (Right) When the data from each zone were pooled among subjects, the significant difference persisted ($p<10^{-3}$) in spite of inter-subject variation in gross IS/OS reflectance. These results illustrate that the IS/OS band over drusen has a lower amplitude than that in healthy retina. Although IQRs overlap in the individual and pooled plots, results are significant due to the large numbers of samples ($6154\le N\le 32927$).

Visual inspection of the B-scans suggested gross differences in IS/OS amplitude between the non-drusen and drusen zones, with non-drusen IS/OS appearing generally brighter. To quantify this difference, the IS/OS amplitude $a_I$ was expressed as the average of five normalized pixels, vertically centered about the segmented IS/OS location, and independent sample t-tests were performed on the resulting non-drusen and drusen samples. The non-drusen $a_I$ was statistically significantly higher than drusen $a_I$ for all subjects individually (Fig. 3, left). The difference was also significant when subject data were pooled (Fig. 3, right), despite intersubject variation in overall B-scan amplitude. High statistical significance ($p<10^{-3}$) was found in spite of the overlapping inter-quartile ranges (IQR) because the number of samples in each group was very high ($6154\le N\le 32927$).

### IS/OS appearance depended on zone and angle of illumination

**Figure 4:**
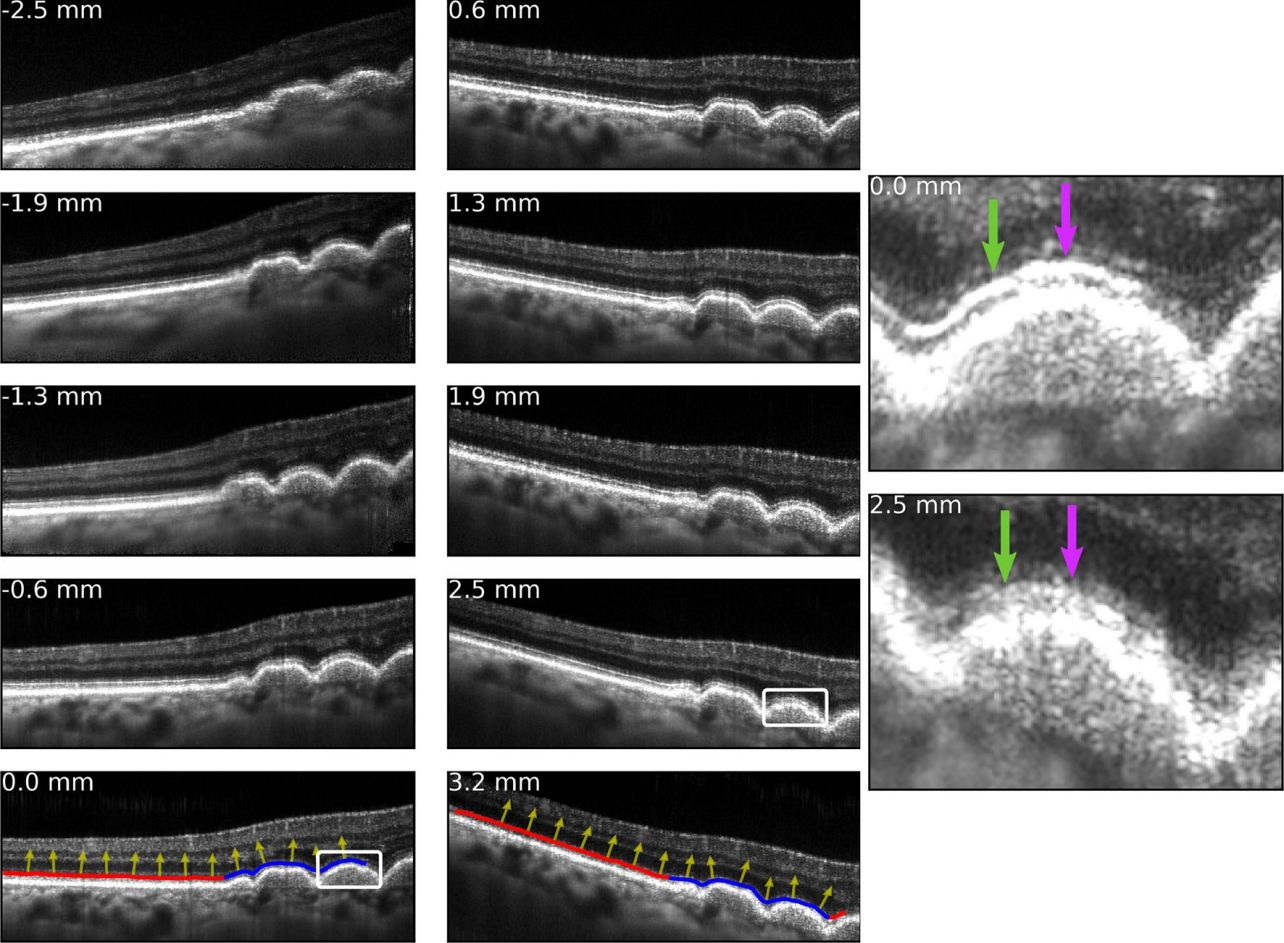
Illustration of the directional effect on OCT image and concept of the analytical approach. The left and center columns are B-scans of a drusen-containing retinal region acquired through different locations in the pupil. The OCT images subtend $6.0^{\circ }$ of visual angle, or approximately 1.8 mm at the retina. At the edges of the pupil the path length variation across the scan angle is maximized, explaining the visible tilt of the retina. In B-scans acquired near the center of the pupil, the retina appears flat. The approximate pupil position, relative to center, is indicated on each image. At this location this subject has several large (200 to $300\,{\upmu }{\mathrm{m}}$) drusen, visible at the right edge of each image. The appearance of the outer retinal bands is affected by angle of illumination, and this is especially visible where the bands are deformed by the underlying drusen (green and purple arrows). Images of one druse acquired at 0.0 mm and 2.5 mm are magnified at the right, illustrating the qualitative directional effects. Incident angle was computed across the scan range, by fitting the IS/OS band and computing the tangent to the fitted curve at all points. This process is illustrated in the bottom images of the left and center columns. Nominally normal and drusen-affected portions of the IS/OS band are colored red and blue, respectively.

Translation of the imaging beam, which induced spatial variation in optical path length, caused the resulting B-scan images to be tilted, as desribed in Fig. 1. A series of images acquired from one subject is shown in Fig. 4, revealing the effect of pupil position on this tilt. Additional angle-dependent differences were qualitatively observable. The overall brightness of the outer retinal bands was highest near the center of the pupil and lowest at the edges. The appearance of portions of IS/OS overlying drusen varied with illumination angle, as illustrated by the purple and green arrows in the magnified drusen images in Fig. 4. Traces through the IS/OS appeared to follow the band closely through both zones. Non-drusen zones are indicated with red lines in Fig. 4 (bottom left and bottom center), while drusen zones are indicated with blue lines. The local tangents computed to this curve (yellow arrows, Fig. 4 bottom left and bottom center) appeared to describe the local IS/OS curvature accurately. The local tangents were used to calculate effective pupil position *x*.

### The amplitude of IS/OS was described well by a Gaussian function of pupil position, with non-drusen and drusen zones having different parameters

**Figure 5:**
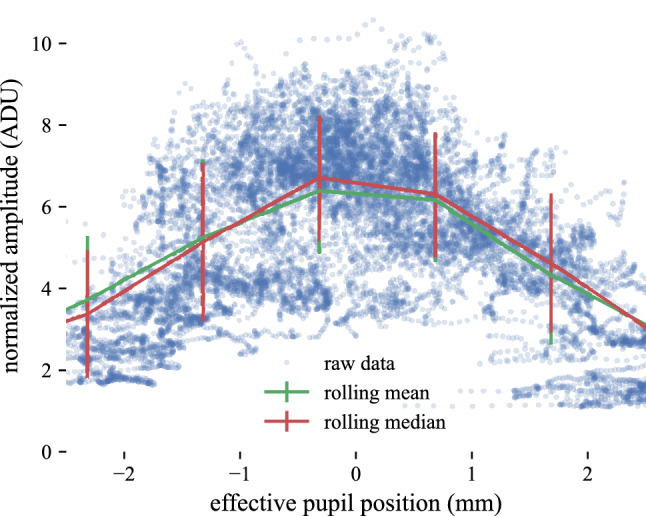
Smoothing IS/OS amplitude. To better visualize the IS/OS reflectance measurements, data were smoothed. In the figure above, the amplitude of the IS/OS from non-drusen retina (subject 2) is plotted as a function of effective pupil position (blue dots). The raw data were smoothed two ways, using a rolling mean (green line) and rolling median (red line). The results of mean- and median-filtering were similar. The standard deviation of the data is illustrated with vertical error bars. An average of 3060 points were used to calculate each median value, and thus the differences in IS/OS amplitude between pupil positions was significant for each 1 mm step. In spite of significant dispersion, a clear relationship can be observed between pupil position and IS/OS amplitude. Sources of dispersion likely include lateral variation in IS/OS reflectivity as well as coherent speckle.

The amplitude of the IS/OS was well-fitted by a Gaussian function of effective pupil position, with significant residual variance (see Fig. 5, blue markers). As a first step in Gaussian modeling of IS/OS directional scattering, we smoothed the data using a discrete median filter. IS/OS amplitude $a_I$ was binned with respect to effective pupil position *x*, using bins of width 2 mm, spaced every 1 mm. A corresponding mean filter was tested as well. Both filters appeared to capture the Gaussian distribution, without significant differences between them (see Fig. 5, green and red lines).

**Figure 6:**
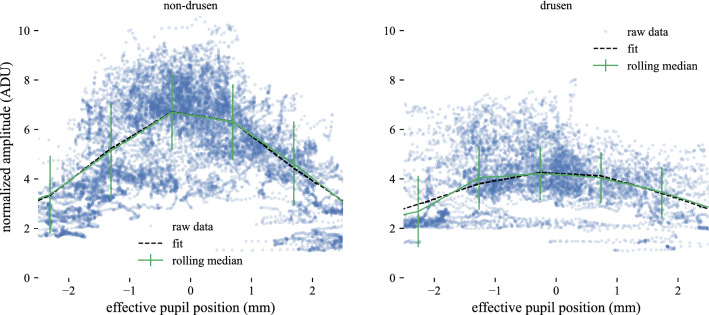
Differences in raw data, smoothed data, and Gaussian fits, between unaffected and drusen-affected IS/OS. When plotted side-by-side, differences in the dependence of IS/OS amplitude on effective pupil position are readily apparent. Raw data are shown with blue circles, while the rolling median and Gaussian fitting function are shown with green lines and black dashed lines, respectively. The Gaussian fits match the smoothed data closely, confirming that the Gaussian equation is a good model for the data in both cases. The raw data appear to be more strongly dependent on effective pupil position in healthy retina than drusen-affected retina, and the amplitude and width of the fitting functions are different, with both parameters being greater in the non-drusen case.

Gaussian fits appeared to match the rolling median closely in all subjects, in both non-drusen and drusen zones. Figure 6 shows the raw data, filtered data, and fit for both zones in subject 2. While the overall difference in $a_I$ between zones (shown in Fig. 3) is visible, the filtered $a_I$ appeared to match the Gaussian fit in both zones.

**Figure 7:**
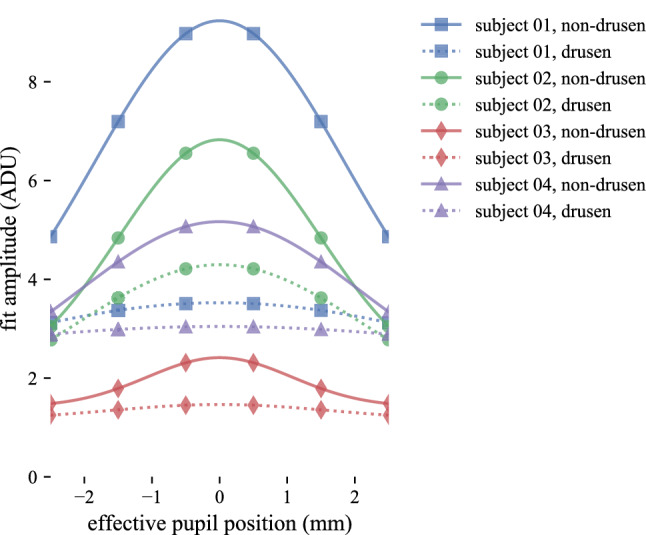
Gaussian models of non-drusen and drusen IS/OS amplitude in all subjects. For each of the four subjects, the Gaussian fits to IS/OS amplitude from non-drusen (blue) and drusen (green) zones are plotted separately. In all four subjects, IS/OS amplitude $a_I$ varied more with effective pupil position *x* in the non-drusen zone than in the drusen zone, suggesting that the optical properties of the photoreceptors in the two zones were different. If the primary cause for reduced visibility of drusen-affected photoreceptors was only disorientation (i.e., pointing away from the pupil), the non-drusen and drusen models would appear similar.

**Figure 8:**
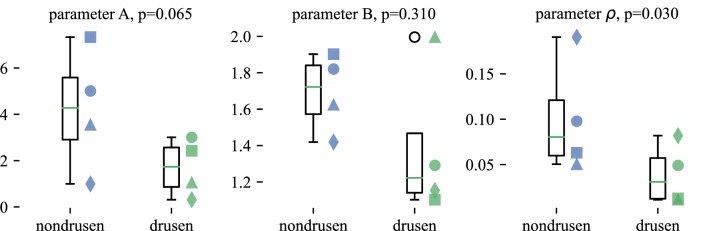
Differences in fitting parameters between non-drusen and drusen zones. After fitting each subject’s non-drusen and drusen IS/OS amplitude to Gaussian functions of effective pupil position, the resulting parameters for Gaussian amplitude, bias, and directionality (width) were pooled across subjects and tested for differences due to zone. While all three parameters had greater values in non-drusen zones compared to drusen zones, only the difference in directionality (Gaussian width) $\rho$ was statistically significant ($p=0.03$). The difference in Gaussian amplitude *A* was marginally significant ($p=0.065$), and the difference in Gaussian bias *B* was not significant ($p=0.31$). The *p* values shown above were somewhat sensitive to parameters in the processing pipeline, particularly the width of the median filter used to smooth the data and the thickness of the segmented region ascribed to IS/OS.

When the models generated by Gaussian fitting were compared, clear differences in their shapes were visible. The non-drusen models had higher average and peak amplitude $a_I$ than the corresponding drusen models, and the non-drusen models appeared less angle-dependent (i.e., flatter) than their drusen counterparts (see Fig. 7). When the model parameters *B*, *A*, and $\rho$ were pooled among subjects and compared between zones, all three were higher in the non-drusen zone, with only $\rho$ showing a statistically significant difference (see Fig. 8). Parameters in the smoothing and fitting procedures (e.g., bin width, optimization method) were not rigorously explored, but choice of these parameters appeared to have some effect on the level of significance of the resulting differences.

## Discussion

The results above imply that the reduced visibility (or reflectivity) of cones overlying drusen is not primarily due to their orientation away from the nominal pupil center. If that were the case, the dependence of IS/OS amplitude on effective pupil position would be similar for non-drusen and drusen zones, since the effective pupil position is derived from the local tissue angle. Drusen-affected IS/OS exhibits some directionality, being most reflective when perpendicular to the imaging beam, as shown in Fig. 6. However, as can be seen in the same figure, the dependence of amplitude on direction is not as strong in drusen-affected IS/OS as in apparently unaffected tissue.

We quantified the directionality of the IS/OS layer using a Gaussian model frequently used to quantify the Stiles–Crawford effect^36^. In this model, the parameter $\rho$ describes the direction-dependence. By calculating $\rho$ in terms of effective pupil position (in millimeters), we were able to produce values that can be compared to other studies of the optical SCE. In non-drusen portions of the retina, we found an average value of $\overline{\rho }=0.10$ ($\sigma _\rho =0.055$). This is in approximate agreement with the values reported by Gao et al. for IS/OS directionality ($\overline{\rho }=0.12$, $\sigma _\rho = 0.036$, $N=4$), using dOCT^23^, and Roorda and Williams ($\overline{\rho }=0.1$, $\sigma _\rho = 0.01$, $N=2$) using AO fundus imaging^37^. Precise calculation of $\rho$ is subject to a number of confounding factors such as coherent interaction of wave guided light and the relative quantities of scattered and wave guided light^36^. We did not make an effort to correct for these factors because we were chiefly interested in the directionality of drusen tissue relative to non-drusen tissue.

In all four subjects, $\rho$ was higher in non-drusen zones than over drusen, as shown in the rightmost plot in Fig. 8, and the difference between the zones was statistically significant ($p=0.03$). Our interpretation of this finding is that the altered appearance of the IS/OS band is not due to disorientation (i.e., pointing). Instead, it appears as though the optical properties of the cones responsible for the optical SCE are altered in cones overlying drusen. The origins of the SCE and optical SCE are not precisely known, although the hypothesis that they arise from the wave guiding behavior of photoreceptors is usually cited as an explanation^38,39^. A detailed survey of this and competing hypotheses—that directionality arises from layered scatterers—can be found in Vohnsen^40^. If the wave guiding hypothesis is correct, one possible contribution to the altered directionality of drusen-affected cones may be multimode propagation. It has been shown that at eccentricities greater than four degrees of visual angle (from the fovea) the inner segments of cone photoreceptors support multiple fiber modes, attributed to the increase in inner segment diameter with eccentricity^41^. Multimode support decreases the directionality factor $\rho$, with increasing numbers of modes leading to flatter profiles. It is also possible that drusen cause a flattening of the cones’ directionality by altering their V number, either by increasing the inner segment diameter or its numerical aperture (e.g., by altering its refractive index). While the results presented above suggest that the optical properties of these cones are affected by the druse, identifying the exact changes would require further experiments, as well as identification of the cause of directional reflectance in cones. Lastly, in AMD the loss of rods is known to precede the loss of cones^42^. In parafoveal locations studied, rods significantly outnumber cones in healthy retinae. We know neither whether the experimental subjects have suffered significant rod loss, nor what the effect of such loss would be on the observed directionality of IS/OS. On the one hand, since rods have significantly flatter Stiles–Crawford directionality (i.e., smaller $\rho$)^43^, one might expect their loss to lead to an *i*ncrease in $\rho$ measured at the IS/OS. On the other hand, rod loss may lead to morphological and/or organizational changes in the remaining cones, potentially decreasing the IS/OS $\rho$.

Our results confirm those of Griffin et al.^32^, who also used dOCT to study drusen. Their results showed that IS/OS amplitude overlying drusen was lower than that between drusen in dry AMD patients. This gross loss of reflectivity can be seen in Fig. 3, within individual subjects and in pooled data. Griffin et al. additionally showed that decentering the beam in the pupil caused a further reduction in IS/OS amplitude in both cases. We observed the same effect, visible, for example, in both zones of subject 2 in Fig. 6 by comparing the IS/OS amplitude at the pupil center (0 mm) with decentered locations (e.g., $-2$ mm and 2 mm). Their study was not designed to measure or model photoreceptor directionality, and the use of just three pupil positions underdetermined the directionality parameter $\rho$. Nevertheless, their observation that decentering the beam in the pupil *decreases* drusen IS/OS reflectivity is consistent with our conclusion that photoreceptor disorientation is not the main reason for reduced photoreceptor visibility; if it were, decentering the beam would have *increased* IS/OS amplitude on the same side of the druse.

Griffin et al. reported a difference in IS/OS amplitude between healthy retinae and apparently unaffected regions in AMD patients (our “non-drusen” zone). As we imaged only AMD patients, we cannot confirm this finding. Their work suggests that a significant fraction of this reduction may be due to decreased directional scattering, since moving the beam away from the center of the pupil results in a greater reduction in IS/OS amplitude in healthy eyes than in non-drusen regions in AMD eyes. We showed notable but not statistically significant reductions in parameters *A* and *B*, which describe the amplitude of the directional and non-directional light scattered from the IS/OS in both non-drusen and drusen zones (see Fig. 8, left and center), which confirms their comparison of the same zones in AMD patients. However, their work did not seek to measure the directionality parameter $\rho$ directly.

Should further work confirm the observed reduction in parameters *A* and *B* in the drusen zone, potential explanations may include outright loss of cones or a change in the reflectivity of the IS/OS. IS/OS reflectivity is thought to be determined by the refractive index mismatch between the inner and outer segments, with possible contributions from structural features seen in that junction using electron microscopy^44^. If the IS/OS reflectivity is altered by drusen, it may be an indication that the refractive index of one or the other compartment has changed, or that mechanical stress due to the druse has altered the structure.

The present study has multiple potential sources of error. First, although a wide range of IS/OS incident angles was measured, more low-angle samples were acquired than high-angle samples. Fitting this raw data to a Gaussian SCE model results in overfitting of data near zero angle and larger error at high angles. The binning, smoothing, and median-filtering described above were used to mitigate this limitation. The filtering approach required selection of two parameters—the binning window width and window step size—without any *a priori* justification. We selected a single set of values that resulted in qualitatively good fits over a wide range of angles and across all subjects. Nevertheless, the goodness of fit appeared to vary with angle for some subjects (see Fig. 5, around $-2$ mm). Filtering the raw data is equivalent to convolution with a *rect* function, which would lead to an underestimate of $\rho$. Since the exact characteristics of the filter were known, numerical deconvolution was possible, but we did not employ it because we suspect the underestimation to be small. Second, in order to factor out variance in IS/OS amplitude due to variations in optical transmission across the pupil, we normalized IS/OS reflectance by the overlying ONL reflectance, which is not thought to be directional. As a result of the ONL band’s low reflectance, it’s SNR is low, leading to increased noise in the IS/OS when normalized to the ONL. Third, despite our best efforts to position the scanning beam, drusen may not have been imaged across the plane containing their maximum height, leading to an added directionality component perpendicular to the scan, not accounted for by our model. If this were the case, the subsequent analysis would underestimate $\rho$ slightly.
